# Biogeographical characteristics of *Schistosoma mansoni* endemic areas in Ethiopia: a systematic review and meta analysis

**DOI:** 10.1186/s40249-021-00864-x

**Published:** 2021-06-07

**Authors:** Keerati Ponpetch, Berhanu Erko, Teshome Bekana, Lindsay Richards, Song Liang

**Affiliations:** 1 Department of Environmental and Global Health, College of Public Health and Health Professions, University of Florida, Gainesville, FL 32610 USA; 2 Aklilu Lemma Institute of Pathobiology, Addis Ababa University, Addis Ababa, Ethiopia; 3 University of Miami Miller School of Medicine, Miami, FL 33136 USA; 4 Emerging Pathogens Institute, University of Florida, Gainesville, FL 32610 USA; 5 Faculty of Public Health and Allied Health Sciences, Ministry of Public Health, Sirindhorn College of Public Health Trang, Praboromarajchanok Institute, Nonthaburi, Thailand

**Keywords:** *Schistosoma mansoni*, Biogeographical characteristic, Systematic review, Ethiopia

## Abstract

**Background:**

In Ethiopia, schistosomiasis is caused by *Schistosoma mansoni* and *S. haematobium* with the former being widespread and more than 4 million people are estimated to be infected by *S. mansoni* annually with 35 million at risk of infection. Although many school- and community-based epidemiological surveys were conducted over the past decades, the national distribution of schistosomiasis endemic areas and associated socio-environmental determinants remain less well understood. In this paper, we review *S. mansoni* prevalence of infections and describe key biogeographical characteristics in the endemic areas in Ethiopia.

**Methods:**

We developed a database of *S. mansoni* infection surveys in Ethiopia through a systematic review by searching articles published between 1975 and 2019 on electronic online databases including PubMed, ScienceDirect, and Web of Science. A total of 62 studies involving 95 survey locations were included in the analysis. We estimated adjusted prevalence of infection from each survey by considering sensitivity and specificity of diagnostic tests using Bayesian approach. All survey locations were georeferenced and associated environmental and geographical characteristics (e.g. elevation, normalized difference vegetation index, soil properties, wealth index, and climatic data) were described using descriptive statistics and meta-analysis.

**Results:**

The results showed that the surveys exhibited a wide range of adjusted prevalence of infections from 0.5% to 99.5%, and 36.8% of the survey sites had adjusted prevalence of infection higher than 50%. *S. mansoni* endemic areas were distributed in six regional states with the majority of surveys being in Amhara and Oromia. Endemic sites were found at altitudes from 847.6 to 3141.8 m above sea level, annual mean temperatures between 17.9 and 29.8 ℃, annual cumulative precipitation between 1400 and 1898 mm, normalized difference vegetation index between 0.03 and 0.8, wealth index score between –68 857 and 179 756; and sand, silt, and clay fraction in soil between 19.1–47.2, 23.0–36.7, and 20.0–52.8 g/100 g, respectively.

**Conclusions:**

The distribution of *S. mansoni* endemic areas and prevalence of infections exhibit remarked environmental and ecological heterogeneities. Future research is needed to understand how much these heterogeneities drive the parasite distribution and transmission in the region.

**Graphic Abstract:**

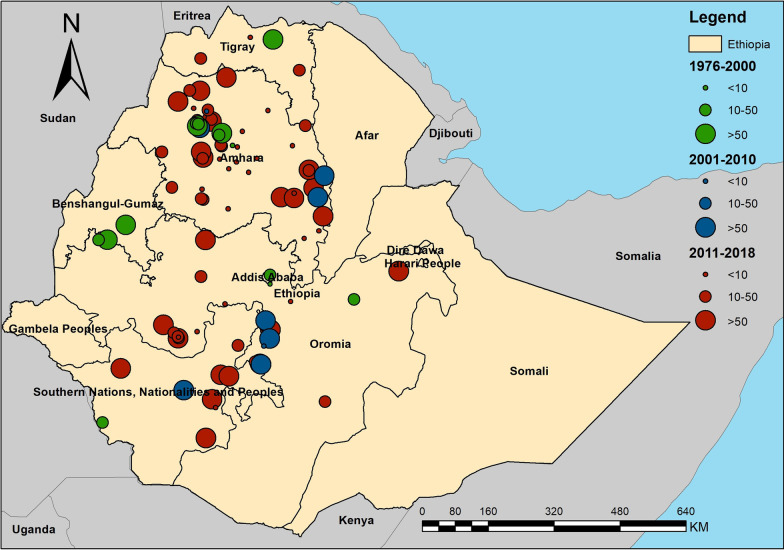

## Background

Schistosomiasis is one of the major public health problems in Ethiopia, causing a high disease burden with an estimate of 5 million people being infected and more than 35 million at risk of infection [1, 2]. The disease is caused by trematode flukes of the genus *Schistosoma.* Schistosomiasis infections in humans may cause acute symptoms such as fever, headache, and myalgia, typically occurring in those who are exposed to schistosome for the first time. The chronic schistosomiasis infection can result in diarrhea, abdominal pain, enlargement of liver, kidney damage, and can cause death in advance cases [3]. Nevertheless, the actual impact of disease or disease burden remains unknown. Children are particularly vulnerable to the disease, and some reports have shown high prevalence of infections among school children [4]. Intestinal schistosomiasis caused by *Schistosoma mansoni* is widely found across the country and prevalence of infection in certain areas can be higher than 85%. For example, Berhe et al. [5] conducted a cross-sectional study in Sille and Worke-Mado villages and found prevalence of *S. mansoni* infection among schoolchildren was 95.0% and 90.6%, respectively. In 2010, the surveys of intestinal schistosomiasis in Kemissie showed *S. mansoni* prevalence of infection to be 89.6% among residents (age 5 to 60 years) [6]. In contrast, urinary schistosomiasis caused by *S. haematobium* is limited to few low-land areas [7].

Numerous surveys have been conducted in different communities in many regions. For example, a high prevalence (40.0–70.0%) of schistosomiasis was reported among schoolchildren in Amhara region [8–10]. Some areas in Oromia were indicated as endemic areas for schistosomiasis with the prevalence ranging between 23.0 and 89.6% [6, 11, 12]. *S. mansoni* prevalence in schoolchildren and residents in Southern Nations, Nationalities, and Peoples’ Region (SNNPR) was reported to be 30.0% [13–15].

The transmission of schistosomiasis involves complex interactions among humans and other possible mammalian hosts, snail intermediate hosts in the freshwater environment, and the schistosome parasites in the hosts and the water environment [16, 17]. The lifecycle of the parasite begins with infectious larvae, cercariae released by infected snails, which act as the intermediate host, can penetrate the skin of humans when they make water contact during daily activities. Cercariae then move to the lungs and liver through blood, and develop to the stage of schistosomula, schistosome worms, that continually produce eggs which are shed to the environment through human excreta. These eggs, once in a freshwater environment, hatch out to miracidia, to infect specific snails to complete the lifecycle [18, 19]. It has long been recognized that environmental factors, such as temperature, precipitation, elevation, soil, and vegetation may limit or promote the distribution of schistosomiasis transmission [20, 21], although some mechanistic understandings of these impacts are largely confined to *S. japonicum* in China and the Philippine [17, 22, 23]. Understanding these impacts, particularly those from modifiable environmental factors [e.g., agricultural practice, water, sanitation and hygiene (WASH), etc.] is important to inform public health surveillance of the disease, environmentally-mediated interventions and sustainable control, as exemplified by the control program in China [24]. Despite sub-Saharan Africa (SSA) bearing the majority of disease burden caused by schistosomiasis, there is a disproportionate lack of studies in the area. As the WHO sets a goal for schistosomiasis endemic countries to achieve elimination as a public health problem by 2030 [25], integrated control (e.g., coupling medical intervention, such as drug treatment, to ecology/environment-oriented management) is likely to play an important role. In addition, the shift of elimination target was largely driven by knowledge gaps in the control effort, in particular those related to implementation and evaluation of the control [26]. Thus, there is a pressing need for improved knowledge on how environmental factors impact schistosomiasis transmission in Africa in general and Ethiopia in particular. In Ethiopia, beyond a study in the early 1980s [27], which described environmental and ecological factors associated with the distribution of *S. mansoni* and *S. haematobium,* there is no systematic work on environmental and geographical characterization of the endemic area at the national scale.

As the first step to address this issue, this study aims to systematically explore biogeographical characteristics of the disease caused by *S. mansoni* in Ethiopia. To do so, a comprehensive database on *S. mansoni* surveys was developed through a systematic review. Also developed was an ecological and environmental database of Ethiopia. A Bayesian model was also used to adjust prevalence of the disease by considering sensitivity and specificity of diagnostic tests used in the surveys included in the systematic review.

## Methods

### Development of database for *S. mansoni* infections

To develop the database on *S. mansoni* infection survey across Ethiopia, a systematic review was conducted using different search strategies to minimize the potential publication bias and obtain literatures related to surveys of schistosomiasis epidemiology in Ethiopia. The keywords: “schistosomiasis” “bilharzia” “*Biomphalaria*”, and “*Schistosoma mansoni*” in conjunction with “Ethiopia” were used for searching articles published and reported in the period from 1975 to 2019 on electronic online databases including PubMed, ScienceDirect, and Web of Science. The inclusion criteria of this review includes publications/reports of any epidemiological surveys or studies on human *S. mansoni* infection, including those from grey literature (e.g., obtained through the assistance of the collaborators from Addis Ababa University), either community or school-based studies. No language constraint was imposed. Publications/reports were not included based on the following exclusion criteria: case reports (e.g., because they cannot provide conclusive evidence of endemic areas), studies conducted on samples outside Ethiopia, studies without the prevalence or infection level of schistosomiasis, and publications of duplicated studies. If the study sites had been surveyed multiple times, the most recent survey was included in the review. We used Egger’s test to examine a publication bias with *P-*value < 0.05 [28].

Relevant data were collected from published articles, including year; name of study site (e.g., regional state, zone and city); geographic coordinates of the study areas; target population; sample size; number of positive individuals; prevalence; infection intensity (e.g., measured through eggs per gram stool); and diagnostic techniques used in the study. For studies without geographic coordinates, Google Earth was used to identify coordinates of study sites. Furthermore, collaborators in Ethiopia helped to identify the locations of surveys that could not be identified by Google Earth.

### Development of database for environmental and ecological factors

Environmental data used in the study were obtained from different sources, as summarized in Table 1. Average temperature and annual precipitation were extracted from WorldClim-Global Climate Data which provides high resolution (approximately 1 km^2^) of interpolated monthly climate data at the global scale [29]. Maximum temperature, minimum temperature and mean precipitation were obtained from the station-based climate dataset for East Africa produced by Gebrechorkos et al. [30]. The ground stations located in Ethiopia were selected and plotted on the Ethiopia boundary map. Means for each daily climate variable were calculated and we then calculated the average values of variables in each station in current periods (1961–2005). Finally, climate data from stations were interpolated using the Empirical Bayesian Kriging technique [31] and used to create a raster file for each variable for further analysis. Elevation data at resolution approximately 250 m updated in 2018 was obtained from CGIAR Consortium for Spatial Information (CGIAR-CSI) [32]. Normalized difference vegetation index (NDVI) data at the global scale was generated by Copernicus Global Land Service using long-term statistics over 1999 to 2017 [33]. Wealth index is the indicator used for characterizing the socioeconomic status of households in African countries and was extracted by Demographic and Health Surveys (DHS) [34]. The wealth index score and cluster coordinates were downloaded from DHS Ethiopia’s survey data in 2016 [35]. Data were then georeferenced and interpolated using the Empirical Bayesian Kriging technique [31]. Soil content data were downloaded from the International Soil Reference and Information Centre (ISRIC) – World Soil information including sand, silt, and clay [36].

**Table 1:** Environmental variables and data sources

Variable	Unit	Resolution	Time period	Source
Elevation	Meters above sea level	250 m		CGIAR Consortium for Spatial Information (CGIAR-CSI) [32]
Wealth index score		250 m	2016	Demographic and Health Surveys (DHS) [34]
Silt	g/100 g	250 m	2008–2012	International Soil Reference and Information Centre (ISRIC) [36]
Sand	g/100 g	250 m	2008–2012	International Soil Reference and Information Centre (ISRIC) [36]
Clay	g/100 g	250 m	2008–2012	International Soil Reference and Information Centre (ISRIC) [36]
NDVI		1 km	1999–2017	Copernicus Global Land Service[33]
Mean temperature	Degree Celsius	1 km	1970–2000	WorldClim[29]
Annual precipitation	mm	1 km	1970–2000	WorldClim [29]
Max temperature	Degree Celsius	250 m	1961–2005	Gebrechorkos SH et al. [30]
Min temperature	Degree Celsius	250 m	1961–2005	Gebrechorkos SH et al. [30]
Mean precipitation	mm	250 m	1961–2005	Gebrechorkos SH et al. [30]

*NDVI* Normalized difference vegetation index

### Statistical analysis and mapping

Geographic coordinates of study sites from all included studies were georeferenced and the spatial distributions were displayed based on administrative and ecological zone maps (East Montane, Eastern, Northeastern, Rift Valley, West Montane, and Western) [37]. Moreover, we examined intestinal schistosomiasis occurrences in the environmental dimension and compared environmental characteristics among different risk levels of communities/study areas: low-risk (prevalence < 10.0%), moderate-risk (prevalence 10% to 50%) and high-risk (prevalence > 50.0%) [38]. Occurrence data were grouped by administrative boundary and ecological zone. Prevalence data were included in the meta-analysis, using a random effect model for pooled estimates of prevalence by grouping unit. Statistical analysis and mapping processes were performed in R studio version 1.3.1073 (RStudio Team, Boston, MA, USA) and ArcGIS version 10.3.1. (ESRI, Redlands, CA, USA).

### Bayesian approach for adjusted prevalence estimation

Since there is no gold standard for detecting schistosome infection, various diagnostic techniques, either alone or in combination, have been used in Ethiopia [39]. Nevertheless, the different sensitivities and specificities of these diagnostic techniques make result comparison difficult and potentially lead to major variations in prevalence and infection intensity estimations [40]. Bayesian inference has commonly been used to infer diagnostic tests when many diagnostic techniques are used in the absence of a gold standard. The Bayesian approach is a powerful tool that can be used to estimate the adjusted prevalence based on initial information including observed prevalence, sensitivity, and specificity of diagnostic test(s) [41]. Sensitivity is the proportion of infected individuals correctly classified as positive. Specificity is the proportion of non-infected individuals correctly classified as negative [42]. Observed prevalence is the proportion of individuals testing positive in the total population. Adjusted prevalence is the proportion of the actual number of infected individuals versus the total number of population [43].

Bayesian estimation of adjusted prevalence can be established by the following equation,

1 $$\pi=\frac{p+SP-1}{SE+SP-1},$$

where $\pi$ = adjusted prevalence, *p* = observed prevalence, $SE$ = sensitivity, $SP$ = specificity.

This approach allows flexibly accounting for uncertainty in the value of *SE* and *SP*. Thus, instead of assuming fixed values, we assume that *SE* can take any possible value in the range of *SE* [44]. Observed prevalence information was obtained from systematic review, whereas sensitivity and specificity of diagnostic techniques were gained from the literatures.

## Results

### General characteristics of the studies

**Fig. 1:**
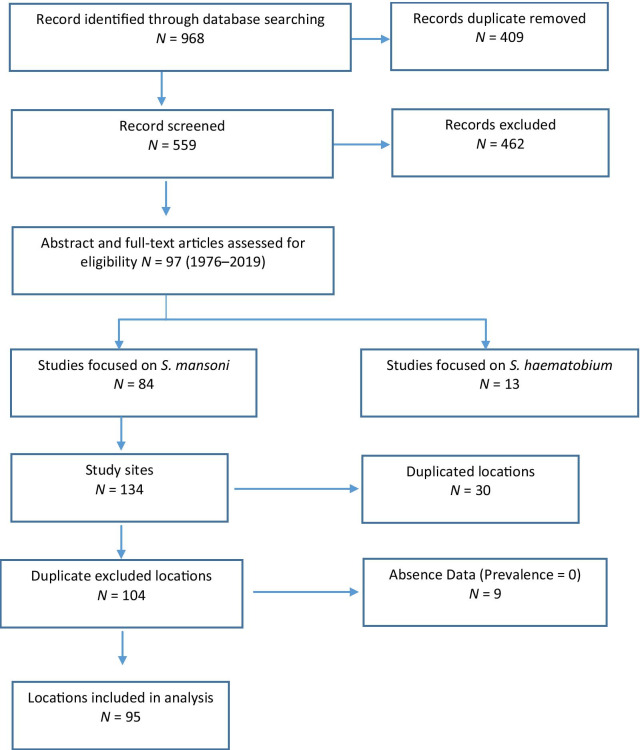
Diagram shows the process of the systematic review for assessing the presence of *Schistosoma mansoni* infection in Ethiopia

Process and results of the systematic review are shown in Fig. 1. The database search identified 968 publications and 409 of them were removed due to duplication. Of the 559 studies screened, 97 articles were assessed based on the abstract and full text, 84 studies were identified to meet the inclusion criteria and were included in review and subsequent analyses. All included articles were based on surveys in 134 sites, and 30 of them had duplicated locations. Among 104 distinct study sites, 9 locations reported zero prevalence of *S. mansoni* infection and were considered as non-endemic areas. Thus, 95 study sites were included in the analysis.

Among 95 localities from 62 studies, 36 studies used a single Kato-Katz; 11 studies used the formalin-ether concentration technique (FECT); and 3 studies used double Kato-Katz. One study used the combined results of six Kato-Katz and triple urine-circulating cathodic antigen (Urine-CCA) to evaluate the diagnostic performance of different Kato-Katz methods and urine-CCA cassette tests [45]. Furthermore, other different approaches were combined to detect *S. mansoni* infection in Ethiopia such as the combination of FECT, single Kato-Katz, and single CCA [46, 47]; the combination of FECT and double Kato-Katz [2]; and the combination of single Kato-Katz and single Sodium acetate-acetic acid-formalin (SAF) solution concentration methods [48, 49] (Table 2).

**Table 2:** Diagnostic tests used for detecting *Schistosoma mansoni* in Ethiopia

Test	Number of study	Number of locations
Single slide Kato-Katz (1-KK)	36	60
Two slides Kato-Katz (2-KK)	3	3
Formalin-ether concentration technique (FECT)	11	20
Single urine circulating cathodic antigen (Single CCA)	1	1
1KK + Single CCA + FECT	2	2
1KK + Sodium acetate-acetic acid-formalin (SAF)	2	2
1KK + FECT + Wet Mount	1	1
1KK + Mini Parasep® solvent-free fecal parasite concentration	1	1
FECT + Wet Mount	2	2
2KK + FECT	1	1
Six slides Kato-Katz + Triple urine circulating cathodic antigen	1	1
Ritchie concentration method	1	1
Total	62	95

### Adjustment of prevalence of infection

**Fig. 2:**
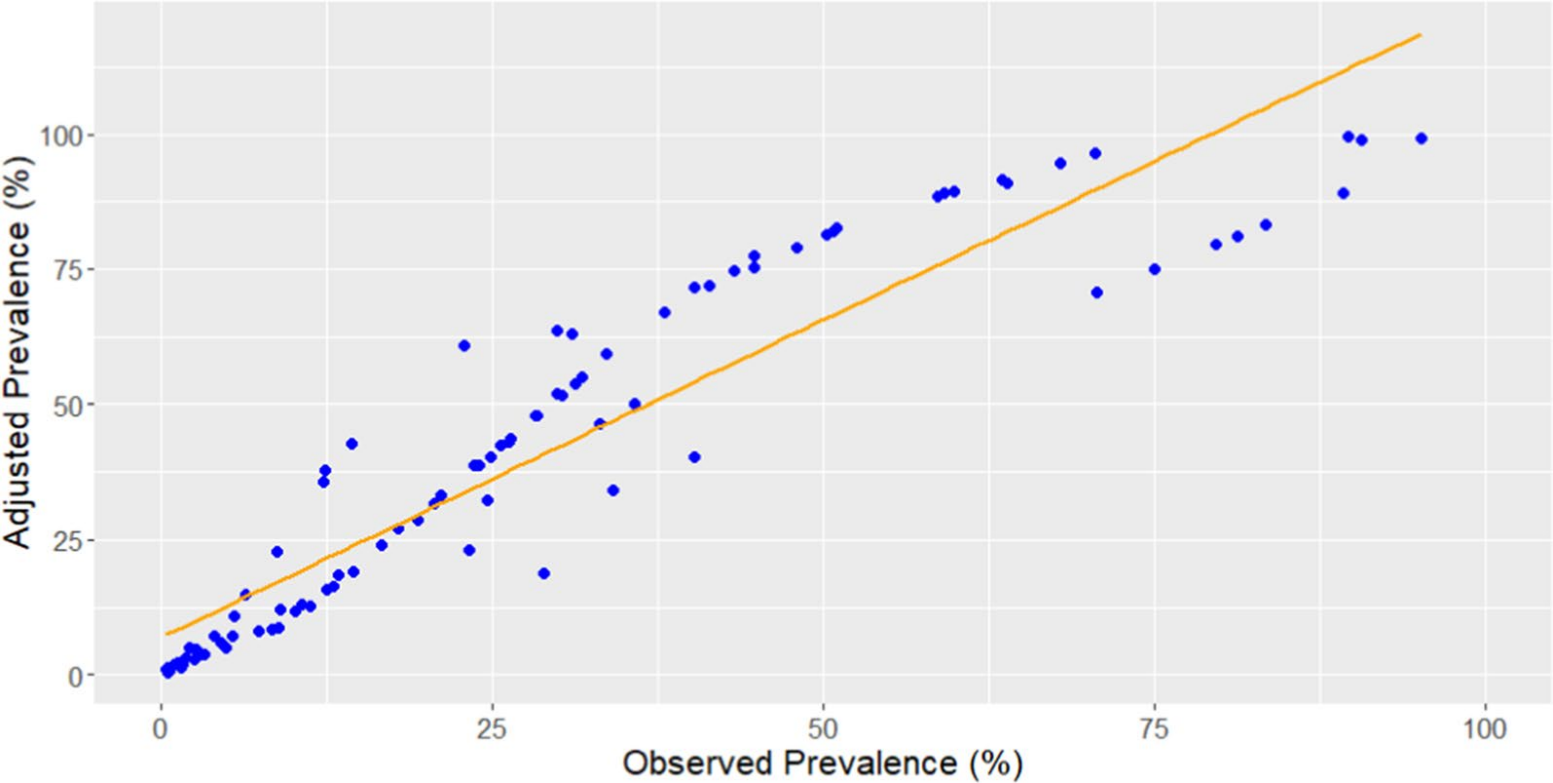
Scatter plot of observed prevalence and adjusted prevalence

Paired *t*-test results indicated that the mean of adjusted prevalence was significantly different from the mean of observed prevalence and the overall prevalence estimate increased by approximately 30.0% after adjustment considering sensitivity and specificity of diagnostic tests (Fig. 2). The surveys exhibited a wide range of prevalence of infections from 0.5% to 99.5%, and 36.8% of the sites had prevalence of infection higher than 50.0%, followed by 34.7% and 28.4% of the sites in the ranges of 10.0–50.0% and < 10.0%, respectively.

### Biogeographical characteristics

**Fig. 3:**
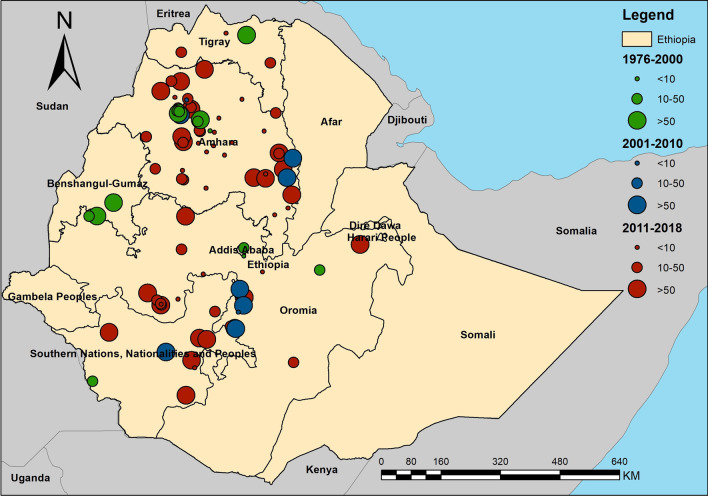
Map shows the distribution and prevalence of *Schistosoma mansoni* infection in Ethiopia

**Fig. 4:**
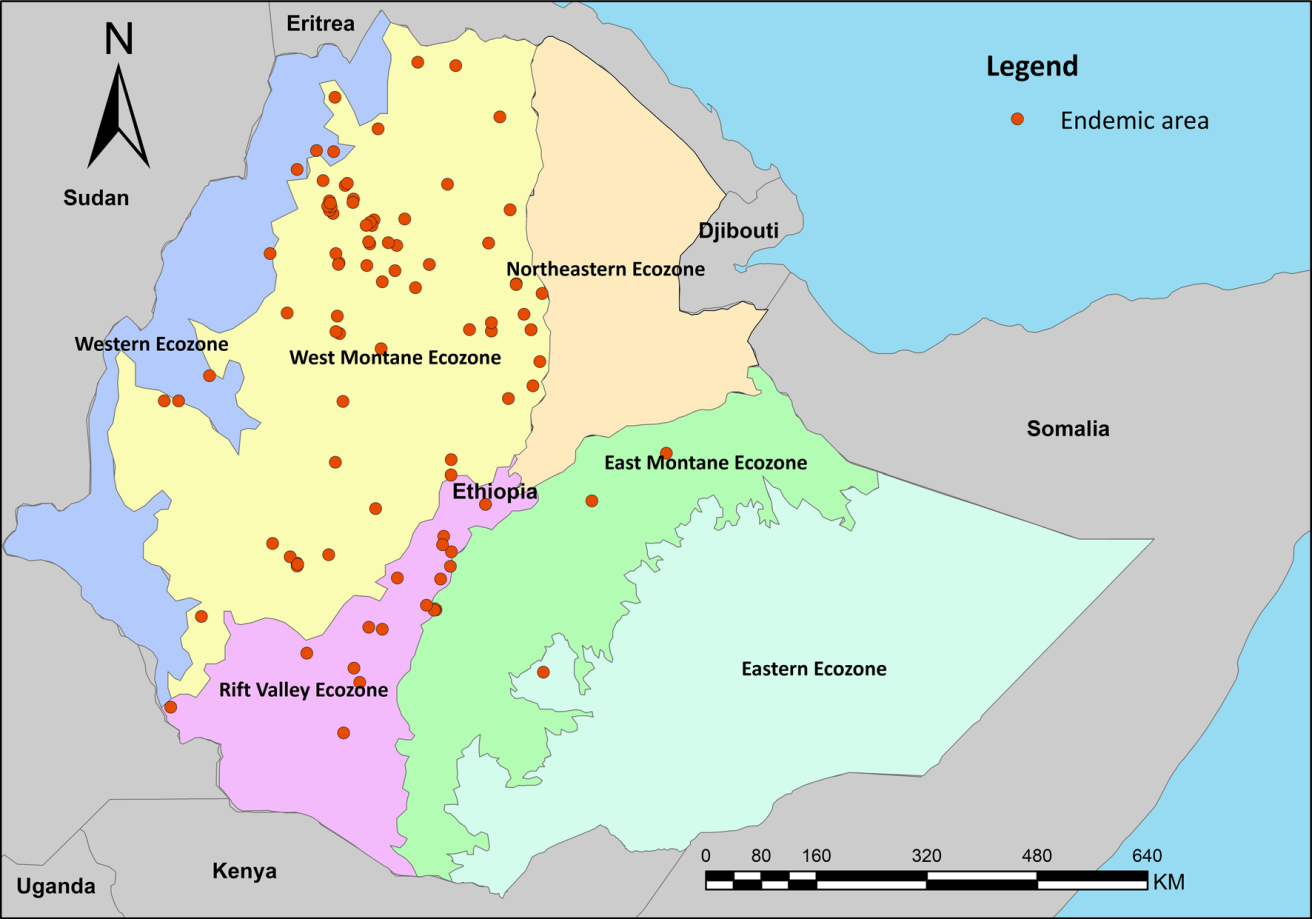
Map shows the distribution *Schistosoma mansoni* infection endemic areas in Ethiopia based on the ecological zone

A total of 95 survey sites were geo-referenced and plotted in administrative boundary and ecological maps of Ethiopia (Figs. 3 and 4). Sites were distributed in six regional states – Southern Nations, Nationalities, and Peoples’ Region (SNNPR); Tigray; Addis Ababa; and Beneshangul Gumuz. A majority of surveys were in Amhara (52 sites) and Oromia (25 sites). In addition, distribution of survey sites based on ecological zone shows that most sites were located in West Montane (68 sites) and Rift Valley ecozones (16 sites). Only one village was surveyed in the Eastern Ecozone.

**Fig. 5:**
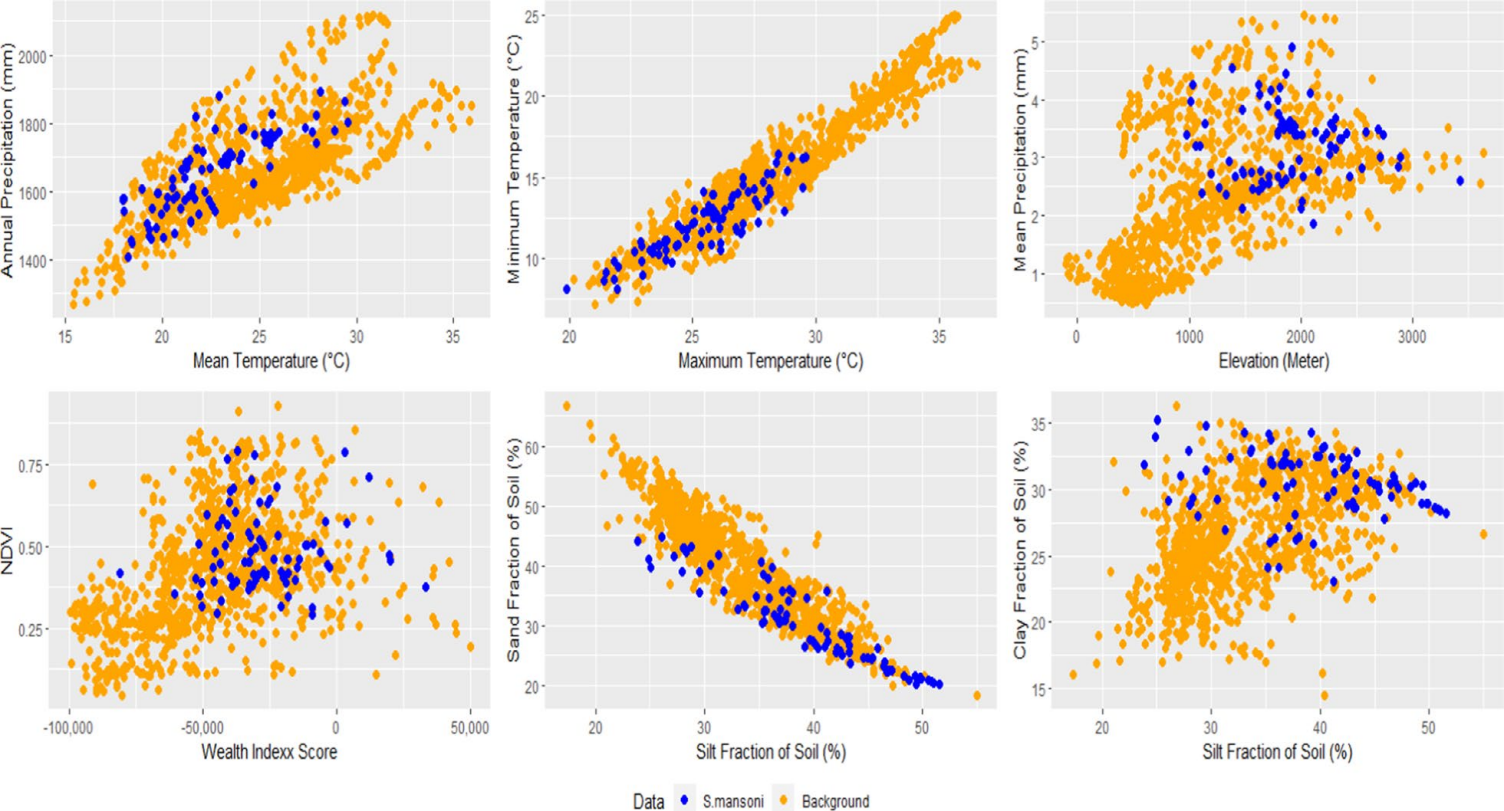
Distribution of *Schistosoma mansoni* infection in the environmental dimensions. Blue dots represent the *S. mansoni* endemic areas and orange dots are the background which are randomly generated to capture the environmental values across the country. *NDVI* Normalized Difference Vegetation Index

**Fig. 6:**
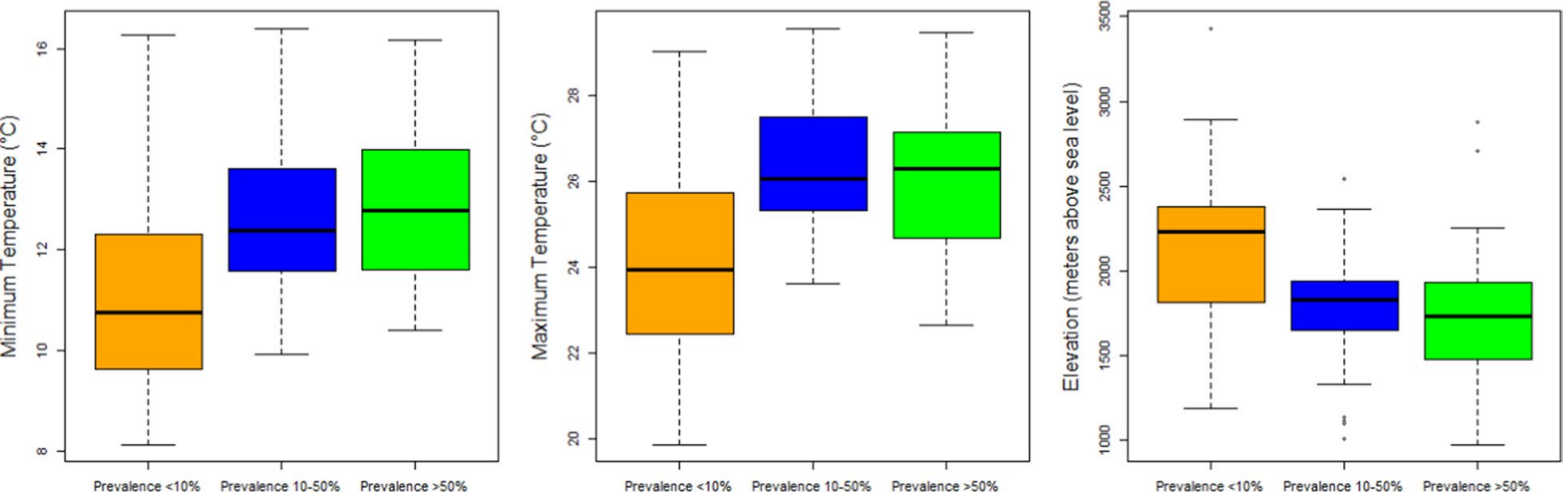
Boxplot shows the distribution of elevation values in prevalence groups

The distribution of schistosomiasis occurrences in environmental dimensions is displayed in Fig. 5. Endemic sites were found at altitudes from 847.6 to 3 141.8 m above sea level, annual mean temperatures between 17.9 ℃ and 29.8 ℃, annual cumulative precipitation between 1400 and 1898 mm, NDVI between 0.03 and 0.8, wealth index score between –68 857 and 179 756, and sand, silt, and clay fraction in soil between 19.1–47.2, 23.0–36.7, and 23.0–52.8 g/100 g, respectively (Table 3). Results showed significant differences in maximum temperature, minimum temperature, and elevation among low-risk, and moderate- and high-risk locations (Fig. 6).

**Table 3:** Comparisons of environmental values between groups of prevalence

Variable	Min–Max	Percentile		Prevalence < 10% (A)		Prevalence 10–50% (B)		Prevalence > 50% (C)		F	Turkey post-hoc test
		5%	95%	Mean	SD	Mean	SD	Mean	SD		
Mean temperature (°C)	17.90–29.80	17.97	28.00	21.82	2.55	22.68	3.19	22.33	2.94	0.65	
Annual precipitation (mm)	1400.00–1898.00	1466.70	1819.00	1635.00	117.88	1654.00	108.09	1652.00	120.65	0.23	
Maximum temperature (°C)	19.88–29.56	21.92	28.74	24.19	2.26	26.28	1.62	26.09	1.78	10.96*	A < B, C
Minimum temperature (°C)	8.12–16.40	9.07	15.52	10.95	1.99	12.69	1.72	12.78	1.58	10.04*	A < B, C
Mean precipitation (mm)	1.87–4.89	2.39	4.21	3.19	0.45	3.28	0.66	33.18	0.67	0.29	
Elevation (Meters above sea level)	847.60–3141.80	1142.26	2557.16	2126.00	467.74	1840.10	363.11	1802.50	372.33	5.74*	A > B, C
NDVI	0.03–0.75	0.16	0.69	0.38	0.10	0.45	0.12	0.42	0.17	1.83	
Silt (g/100 g)	22.98–36.73	26.30	33.85	31.01	1.81	29.97	2.39	30.33	3.02	1.30	
Sand (g/100 g)	19.11–47.22	20.24	43.10	29.94	6.06	29.50	8.64	32.43	6.72	1.58	
Clay (g/100 g)	22.89–52.81	25.80	51.44	39.05	5.99	40.53	8.90	37.23	7.21	1.63	
Wealth index score	−68 857.00–179 756.00	−51 503.92	21 657.24	−22 877	46 302.59	−11 783.00	43 861.37	−25 735.00	20 550.91	1.91	

* means significant difference between A, B and C; *NDVI* Normalized Difference Vegetation Index

### Meta-analysis

Substantial heterogeneities in prevalence of infections were found across the country with an overall prevalence of 31.8% (95% *CI*: 22.9, 42.2) (Tables 3, 4). Tigray was the region with the highest prevalence (prevalence 73.0%, 95% *CI*: 36.5, 92.7, and *I*^*2*^ = 99.4%), followed by SNNPR (prevalence 51.2%, 95% *CI*: 27.7, 74.2, and *I*^*2*^ = 99.6%), Amhara (prevalence 30.0%, 95% *CI*: 18.3, 45.0, and *I*^*2*^ = 99.5%), and Oromia (prevalence 20.4%, 95% *CI*: 10.9, 34.9, and *I*^*2*^ = 99.7%). *I*^*2*^ was not estimated for Addis Ababa and Beneshangul Gumaz because there was only one study conducted in each region.

**Table 4:** Pooled estimates of schistosomiasis prevalence in Ethiopia by administrative boundary and ecological zone

Group		No. of studies	Prevalence (95% *CI*) (%)	Sample	Heterogeneity test (*I*^*2*^) (%)
Overall		95	31.77 (22.87–42.23)	49 075	99.7
Administrative boundary	Addis Ababa	1	11.68	197	
	Amhara	52	29.96 (18.27–45.01)	27 968	99.7
	Beneshangul Gumaz	1	63.43	176	
	Oromia	25	20.44 (10.95–34.93)	14 509	99.4
	SNNPR	11	51.22 (27.74–74.17)	4629	99.6
	Tigray	5	72.99 (36.51–92.70)	1794	99.4
Ecozone	East Montane	5	53.22 (30.78–74.43)	5236	98.5
	Eastern	1	38.10	340	
	Rift Valley	16	48.18 (23.52–73.77)	5494	99.5
	West Montane	68	25.48 (16.56–37.08)	34 350	99.4
	Western	6	56.37 (25.28–83.14)	3845	99.3

Among ecological zones, the highest prevalence was observed in Western Ecozone (prevalence 56.4%, 95% *CI*: 25.3, 83.1, and *I*^*2*^ = 99.3%) followed by East Montane Ecozone (prevalence 53.2%, 95% *CI*: 30.8, 83.1, and *I*^*2*^ = 99.5%), and Rift Valley Ecozone (prevalence 48.2%, 95% *CI*: 23.5, 73.8, and *I*^*2*^ = 99.5%). West Montane Ecozone, for which 68 studies were conducted, had the lowest prevalence at 25.5% (95% *CI*: 16.6, 37.1), and *I*^*2*^ = 99.4%.

## Discussion

In this study, a systematic review of *S. mansoni* infections in the country was performed, including 62 studies that were conducted in 95 sites and reported between 1976 and 2019. The first review regarding schistosomiasis in Ethiopia was published in 1988 with aims to compile the data from epidemiological surveys between 1961 and 1986 and to discuss the possible influence of climate, elevation, ecology of snail, and economic condition on the spread of schistosomiasis in Ethiopia [27]. In addition, the most recent review was carried out in 2014, focusing on the challenges of schistosomiasis prevention and control programs in Ethiopia such as the possible impact of the irrigation development, current interventions, and national policies [4].

There is no gold standard for diagnosing schistosomiasis [50], and various techniques have been developed and used to detect parasite eggs including Kato-Katz and the formal-ether concentration techniques. However, these approaches are subject to variations in their sensitivity, specificity, and limitations [46]. Estimating “adjusted” diagnostic results by considering these parameters has been widely practiced in applications and many statistical approaches have been developed for the purpose. The Bayesian approach is widely used and is a valuable tool providing solutions to combine the results from different diagnostic techniques and to standardize the test results based on the sensitivity and specificity of each diagnostic test [51]. Thus, the Bayesian approach is a useful tool to adjust schistosomiasis prevalence data gained from different diagnostic techniques. The study is among the first to develop nonbiased population-based estimate of schistosomiasis infection by accounting for sensitivity and specificity of diagnostics used in the population surveys.

In this study, the biogeographic characteristics of endemic areas based on known data from the systematic review were extracted and discussed. Our results show that the majority of endemic areas are located in West Montane and Rift Valley ecozones, indicating a high rate of infection in villages around Lake Tana and in the Rift Valley areas near Ziway and Abaya lakes, which was in agreement with reports from three decades ago [27]. Temperature is an important determinant of transmission of schistosomiasis, influencing parasite development [52] and lifecycle of snail intermediate hosts [53]. Endemic areas of *S. mansoni* in our study had temperature ranges between 17.9 and 29.8 degrees Celsius. This temperature range differs little from temperature values of a 2001 study in Ethiopia and East Africa which reported a suitable temperature range at 20.0 to 30.0 degree Celsius [54]. The temperature range from our finding falls within the range of optimal temperature for *Biomphalaria pfeifferi*, the *S. mansoni* snail intermediate host [55]. Precipitation also plays a critical role in schistosomiasis transmission as it affects water flow velocities and water temperature which in turn, impact the development of parasites in the environment and in snail intermediate hosts [52]. Elevation is another possible factor to identify potential distribution of disease. Approximately 90% of our study sites were located at elevation between 1142 and 2257 m above sea level, which again, is largely similar from a previous review which suggested the range of suitable altitude at 1300 and 2200 m above sea level [27]. In addition, our results showed that the wealth index score could be used to identify the group of people with a high risk of infection in Ethiopia. Most endemic areas are where the wealth index scores are lower than zero. This indicates a poor population lacking appropriate household assets and services. This indicator was used to determine subgroups to be (re)treated and (re)examined, and also used to inform new treatment strategies in Uganda [56]. Soil is also an important factor associated with the transmission of schistosomiasis as it is closely related to ecology of snail intermediate hosts as well as land use and agricultural practices, which have long been recognized as important factors in studies of *S. japonicum* in China and the Philippines [22]. This review suggests that schistosomiasis endemic areas in Ethiopia are largely in areas where the soil’s silt and clay contents are higher than 22.0%. These two soil components support the growth of aquatic vegetation that makes the area more suitable for snail intermediate hosts [57].

We also explored biogeographical characteristics associated with *S. mansoni* endemic areas in Ethiopia, from both environmental and geographic dimensions. The distribution of endemic areas in the country exhibits substantial heterogeneities in temperature, precipitation, and elevation with clear ecological/environmental “limits” across the country (Fig. 3). Ethiopia has diverse ecological and environmental landscapes – lakes, high mountains and plateau, and valley (Rift Valley)—and *S. mansoni* endemic areas are reported in most of the landscapes. This is interesting, in comparison with some schistosomiasis endemic areas elsewhere in Africa where the endemic environment is relatively homogenous [27], and it has important public health implications from two perspectives. First, tailored control strategies focused on specific transmission ecology, as exemplified by some successes with *S. japonicum* [58, 59], could be potentially important for sustainable control of schistosomiasis in Ethiopia. Second, the environmental/ecological diversity of endemic areas also raise concerns over the potential impact of environmental change (e.g., urbanization, water resource projects, agricultural development, and climate change) on schistosomiasis transmission and distribution in Ethiopia [10, 60].

The study has some limitations related to the occurrence data. The occurrence data, as reflected by the endemic sites included in this study, were largely extracted from cross-sectional surveys through a systematic review. These study sites were selected by the researchers based on their experience, previous surveys/surveillance, among others, leading to inclusion of potentially ‘incomplete’ suitable sites of schistosomiasis and influencing biogeographical characteristics of *S. mansoni* endemic areas. There are some areas where schistosomiasis transmission may occur or environmental factors may meet the ecological requirement of disease transmission but had not been surveyed. This limitation can be addressed in part by new studies and/or surveillance from unknown areas, model projections (e.g., based on niche models from our accompanying work) to inform suspected areas for the disease transmission, or a combination of both. This effort would be instrumental for schistosomiasis surveillance systems in Ethiopia.

## Conclusions

The distribution of *S. mansoni* endemic areas and prevalence of infections exhibit remarked environmental and ecological heterogeneities. It is well recognized that future research, particularly focusing on a mechanistic understanding of how the environmental change will influence transmission, is much needed in the country. Further knowledge in these areas will certainly help to inform improved public health surveillance and control programs in Ethiopia.

## Data Availability

The dataset analysed during the current study will be available in the following repositories (Open Science Framework [https://osf.io]) or upon request to the authors.

## Abbreviations

SNNPR: Southern Nations, Nationalities, and Peoples’ Region
NDVI: Normalized Difference Vegetation Index
DHS: Demographic and Health Survey
SE: Sensitivity
SP: Specificity
FECT: Formalin-ether concentration technique
CCA: Circulating Cathodic Antigen
SAF: Sodium acetate–acetic acid-formalin
